# RNA-seq and Tn-seq reveal fitness determinants of vancomycin-resistant *Enterococcus faecium* during growth in human serum

**DOI:** 10.1186/s12864-017-4299-9

**Published:** 2017-11-21

**Authors:** Xinglin Zhang, Vincent de Maat, Ana M. Guzmán Prieto, Tomasz K. Prajsnar, Jumamurat R. Bayjanov, Mark de Been, Malbert R. C. Rogers, Marc J. M. Bonten, Stéphane Mesnage, Rob J. L. Willems, Willem van Schaik

**Affiliations:** 1 College of Biosystems Engineering and Food Science, Zhejiang University, Hangzhou, 310058 China; 2 Department of Medical Microbiology, University Medical Center Utrecht, 3584CX Utrecht, the Netherlands; 3 Krebs Institute, University of Sheffield, Sheffield, S10 2TN United Kingdom; 4 Institute of Microbiology and Infection, College of Medical and Dental Sciences, The University of Birmingham, Birmingham, B15 2TT United Kingdom

**Keywords:** *Enterococcus faecium*, Transcriptome, Transposon mutant library screening, Nucleotide biosynthesis, Carbohydrate metabolism, Virulence, Zebrafish

## Abstract

**Background:**

The Gram-positive bacterium *Enterococcus faecium* is a commensal of the human gastrointestinal tract and a frequent cause of bloodstream infections in hospitalized patients. The mechanisms by which *E. faecium* can survive and grow in blood during an infection have not yet been characterized. Here, we identify genes that contribute to growth of *E. faecium* in human serum through transcriptome profiling (RNA-seq) and a high-throughput transposon mutant library sequencing approach (Tn-seq).

**Results:**

We first sequenced the genome of *E. faecium* E745, a vancomycin-resistant clinical isolate, using a combination of short- and long read sequencing, revealing a 2,765,010 nt chromosome and 6 plasmids, with sizes ranging between 9.3 kbp and 223.7 kbp. We then compared the transcriptome of *E. faecium* E745 during exponential growth in rich medium and in human serum by RNA-seq. This analysis revealed that 27.8% of genes on the *E. faecium* E745 genome were differentially expressed in these two conditions. A gene cluster with a role in purine biosynthesis was among the most upregulated genes in *E. faecium* E745 upon growth in serum. The *E. faecium* E745 transposon mutant library was then used to identify genes that were specifically required for growth of *E. faecium* in serum. Genes involved in de novo nucleotide biosynthesis (including *pyrK_2, pyrF, purD, purH*) and a gene encoding a phosphotransferase system subunit (*manY_2*) were thus identified to be contributing to *E. faecium* growth in human serum. Transposon mutants in *pyrK_2, pyrF, purD, purH* and *manY_2* were isolated from the library and their impaired growth in human serum was confirmed. In addition, the *pyrK_2* and *manY_2* mutants were tested for their virulence in an intravenous zebrafish infection model and exhibited significantly attenuated virulence compared to *E. faecium* E745.

**Conclusions:**

Genes involved in carbohydrate metabolism and nucleotide biosynthesis of *E. faecium* are essential for growth in human serum and contribute to the pathogenesis of this organism. These genes may serve as targets for the development of novel anti-infectives for the treatment of *E. faecium* bloodstream infections.

**Electronic supplementary material:**

The online version of this article (10.1186/s12864-017-4299-9) contains supplementary material, which is available to authorized users.

## Background

Enterococci are commensals of the gastrointestinal tract of humans and animals, but some enterococcal species, particularly *E. faecium* and *E. faecalis,* are also common causes of hospital-acquired infections in immunocompromised patients [1]. While *E. faecalis* has been recognized as an important nosocomial pathogen for over a century, *E. faecium* has emerged as a prominent cause of hospital-acquired infections over the last two decades [2]. Since the 1980s, *E. faecium* acquired resistance to multiple antibiotics, including β-lactams, aminoglycosides and finally, to the glycopeptide vancomycin [3]. Nosocomial infections are almost exclusively caused by a specific sub-population of *E. faecium,* termed clade A-1, which has emerged from a background of human commensal and animal *E. faecium* strains [4]. Strains in clade A-1 carry genetic elements that are absent from animal or human commensal isolates and which contribute to gut colonization or pathogenicity [5–9]. Clade A-1 *E. faecium* strains are rarely found in healthy individuals but can colonize the gut of immunosuppressed, hospitalized patients to high-levels. These strains can then cause infections by direct translocation from the gut into the bloodstream [10–12]. In addition, due to faecal contamination of the skin in hospitalized patients, the use of intravenous catheters is another risk factor for the introduction of *E. faecium* into the bloodstream [3, 13, 14]. Currently, *E. faecium* causes approximately 40% of enterococcal bacteremias. Due to the accumulation of antibiotic resistance determinants in clade A-1 strains, *E. faecium* infections are more difficult to treat than infections caused by *E. faecalis* or other enterococci [15–17]. To cause bloodstream infections, *E. faecium* needs to be able to survive and multiply in blood, but the mechanisms by which it can do so, have not yet been studied. To thrive in the bloodstream, an opportunistic pathogen has to evade host immune mechanisms and to adjust its metabolism to an environment that is relatively poor in nutrients [18].

To identify genes that are conditionally essential in bacteria, high-throughput screening methods for transposon mutant libraries have been developed and optimized for many different bacterial species [19, 20]. To perform high-throughput functional genomics in ampicillin-resistant, vancomycin-susceptible clinical *E. faecium* strains, we previously developed a microarray-based transposon mutagenesis screening method which was used to identify genes involved in the development of endocarditis [7], resistance to ampicillin [21], bile [22] and disinfectants [23]. However, microarray-based methods for transposon mutant library screening are limited in their accuracy and can only be used in strains for which the microarray was designed. To address these limitations, several methods, including Tn-seq [24] and TraDIS [25], which are based on high-throughput sequencing of the junctions of the transposon insertion sites and genomic DNA, have been developed [26].

In this study, we set-up Tn-seq in the clinical *E. faecium* isolate E745 to identify genes that contribute to survival and growth in human serum. In addition, we determined the transcriptional response of *E. faecium* E745 in that same environment. Finally, we substantiated the role of two *E. faecium* genes that contribute to growth in serum and in virulence, in a zebrafish model of infection. Collectively, our findings show that metabolic adaptations are key to *E. faecium* growth in serum and contribute to virulence.

## Results

### The complete genome sequence of *E. faecium* E745

**Fig. 1:**
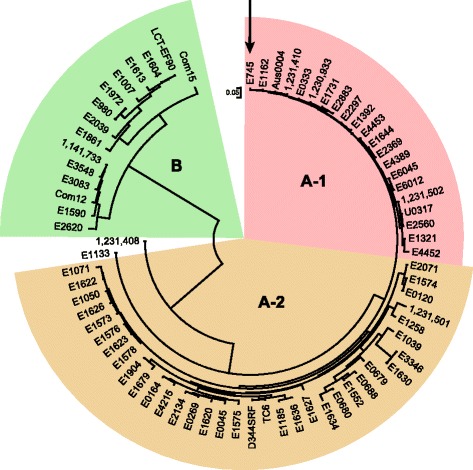
Maximum likelihood phylogenetic tree of *E. faecium.* The phylogenetic tree was based on a core genome alignment of 1,545,750 positions that was generated by ParSNP [61]. The tree includes the *E. faecium* E745 genome sequence generated in this study and the 72 *E. faecium* whole genome sequences described in Lebreton et al. [4]. The tree was visualized and mid-point rooted using MEGA 7.0.26 [62]. The different *E. faecium* clades are indicated. The position of *E. faecium* E745 in the phylogenetic tree is highlighted by an arrow

In this study, we implemented RNA-seq and Tn-seq analyses in *E. faecium* strain E745, an ampicillin- and vancomycin-resistant clinical isolate. *E. faecium* E745 was isolated from a rectal swab of a hospitalized patient as part of routine surveillance during an outbreak of VRE in the nephrology ward of a Dutch hospital in 2000 [27, 28]. To allow the application of RNA-seq and Tn-seq in *E. faecium* E745, we first determined the complete genome sequence of this strain through a combination of short-read Illumina sequencing and long-read sequencing on the RSII Pacific Biosciences and Oxford NanoPore’s MinION systems. This resulted in a closed chromosomal sequence of 2,765,010 nt and 6 complete plasmids sequences, with sizes ranging between 9.3 kbp and 223.7 kbp (Additional file 1). Taken together, the chromosome and plasmids have 3095 predicted coding sequences. Phylogenetic analysis of the core genome of E745 and a set of 72 genomes representing global *E. faecium* diversity [4], showed that *E. faecium* E745 is a clade A-1 strain (Fig. 1). The E745 chromosome contains a pathogenicity island with the *esp* gene, which encodes a 207-kDa surface protein that is involved in biofilm formation and infection [6, 29, 30]. The vancomycin resistance genes of *E. faecium* E745 are of the *vanA* type [31] and are carried on the 32.4-kbp plasmid pE745-2. Additional antibiotic resistance genes in the *E. faecium* E745 genome are the trimethoprim resistance gene *dfrG* [32], which is located on plasmid pE745-6, and the chromosomally encoded macrolide resistance gene *msrC* [33].

### Transcriptome of *E. faecium* E745 during growth in rich medium and in human serum

**Fig. 2:**
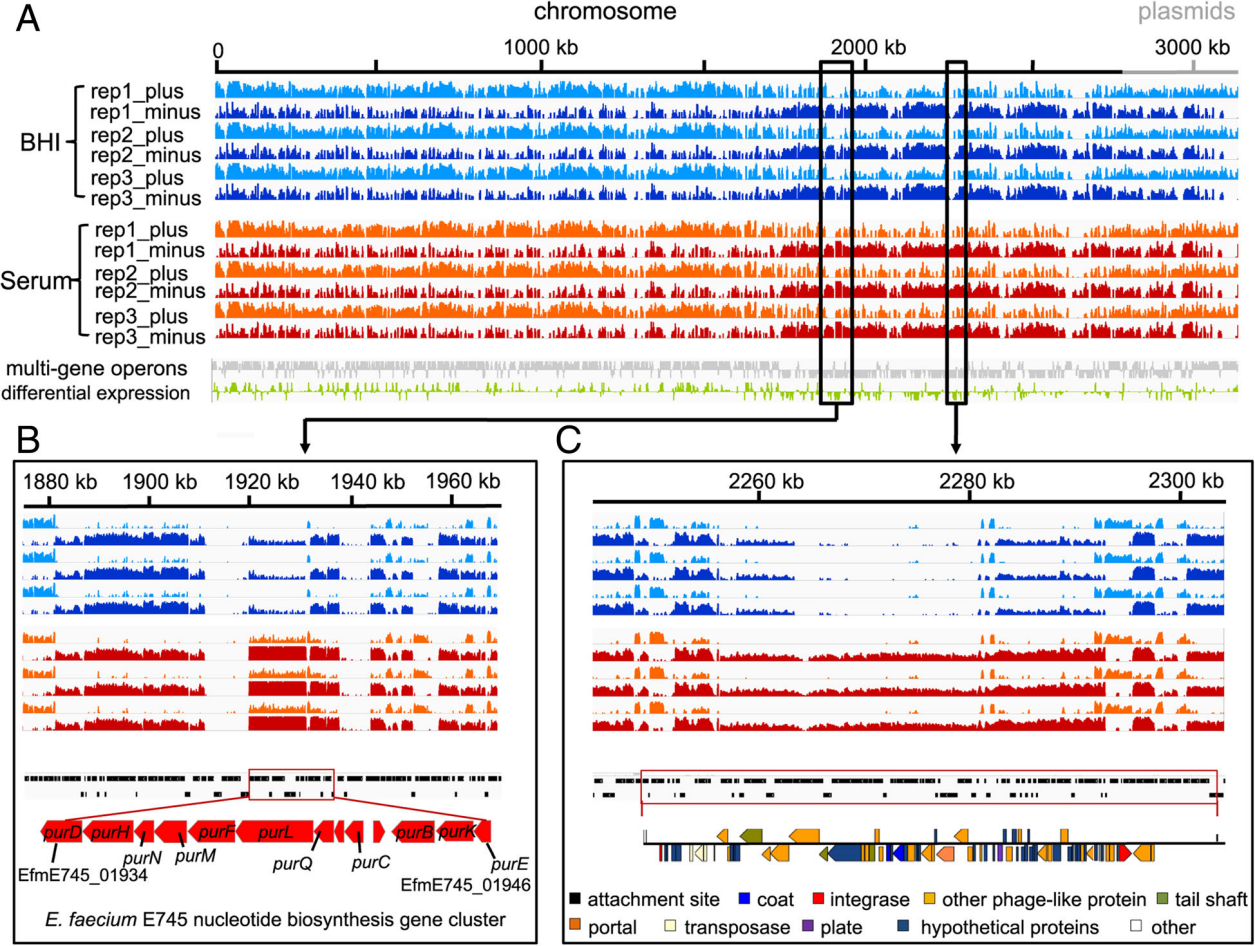
Transcriptome analysis of *E. faecium* E745. Coverage plots of RNA-seq data aligning to chromosome and plasmid DNA are shown in panel **a**. The y-axis of each track indicates reads coverage and is represented on a log scale, ranging from 0 to 10,000. The x-axis represents the genomic location. Light blue (BHI) or orange (serum) tracks correspond to sequencing reads aligned to the plus strand of the replicon, and dark blue (BHI) or dark red (serum) tracks correspond to sequencing reads aligned to the minus strand of the replicon. The grey track corresponds to multi-gene operons. The green track corresponds to differentially expressed genes (BHI vs serum), with the height of the green bars indicative of differential expression. In panels **b** and **c**, two serum-induced regions are shown, i.e. a gene cluster involved in nucleotide biosynthesis (panel **b**) and a prophage (panel **c**). The RNA-seq experiments were performed using three biological replicates

After confirming that serum can support the growth of *E. faecium* E745, though at a lower growth rate than the rich medium BHI (Additional file 2), the transcriptional profile of E745 was determined by RNA-seq during exponential growth in BHI and in heat-inactivated human serum. A total of 99.9 million (15.6–17.6 million per sample) 100 bp paired-end reads were successfully aligned to the genome, allowing the quantification of rare transcripts (Fig. 2). A total of 3217 transcription units were identified, including 651 predicted multi-gene operons, of which the largest contains 22 genes (Fig. 2a and Additional file 3).

A comparative analysis of the E745 transcriptome during growth in BHI and in human serum, showed that 860 genes exhibited significantly (*q* < 0.001 and a fold change in expression of >2 or <0.5 between cultures grown in BHI versus heat-inactivated serum) different expression between these conditions (Additional file 4). Among the genes with the highest difference in expression between growth in serum and in rich medium, we identified a gene cluster with a role in purine biosynthesis (Fig. 2b). In addition, we found a 58.4 kbp prophage-like gene cluster that exhibited higher expression in E745 during growth in serum (Fig. 2c). Analysis of this phage sequence against genome sequences deposited at NCBI Genbank revealed that an essentially identical element (with 99% nucleotide identity) could be identified in *E. faecium* ATCC 700221 but not in other genome sequences of *E. faecium* or other bacteria.

To confirm the RNA-seq analysis, we independently determined expression levels of eight genes during growth in serum versus growth in BHI by qPCR (Additional file 5). RNA-seq and qPCR data were highly concordant (r^2^ = 0.98).

### *E. faecium* E745 genes required for growth in human serum

A *mariner*-based transposon mutant library was generated in *E. faecium* E745 and Tn-seq [24] was performed on ten replicate transposon mutant libraries (after overnight growth in BHI at 37 °C), resulting in an average of 15 million Tn-seq reads for each library. To analyze the Tn-seq data, we divided the E745 genome in 25-nt windows. Of a total of 110,601 25-nt windows, 49,984 (45%) contained one or more sequence reads. No positional bias was observed in the transposon insertion sites in the chromosome and plasmids of *E. faecium* E745 (Additional file 6).

In order to identify genes that contribute to growth of *E. faecium* E745 in human serum, we performed Tn-seq on cultures of the *E. faecium* E745 transposon mutant libraries upon growth in rich medium (BHI) and in human serum. The human serum was either used natively, or was heat-treated to inactivate the complement system [34]. Minor differences were observed among conditionally essential genes between the experiments performed in native human serum or heat-inactivated human serum (Additional file 7) and the following results correspond to the experiments obtained with heat-inactivated serum. This condition was chosen because it may be a more reproducible in vitro environment*,* particularly since the interaction between the complement system and Gram-positive bacteria remains to be fully elucidated [35, 36].

**Fig. 3:**
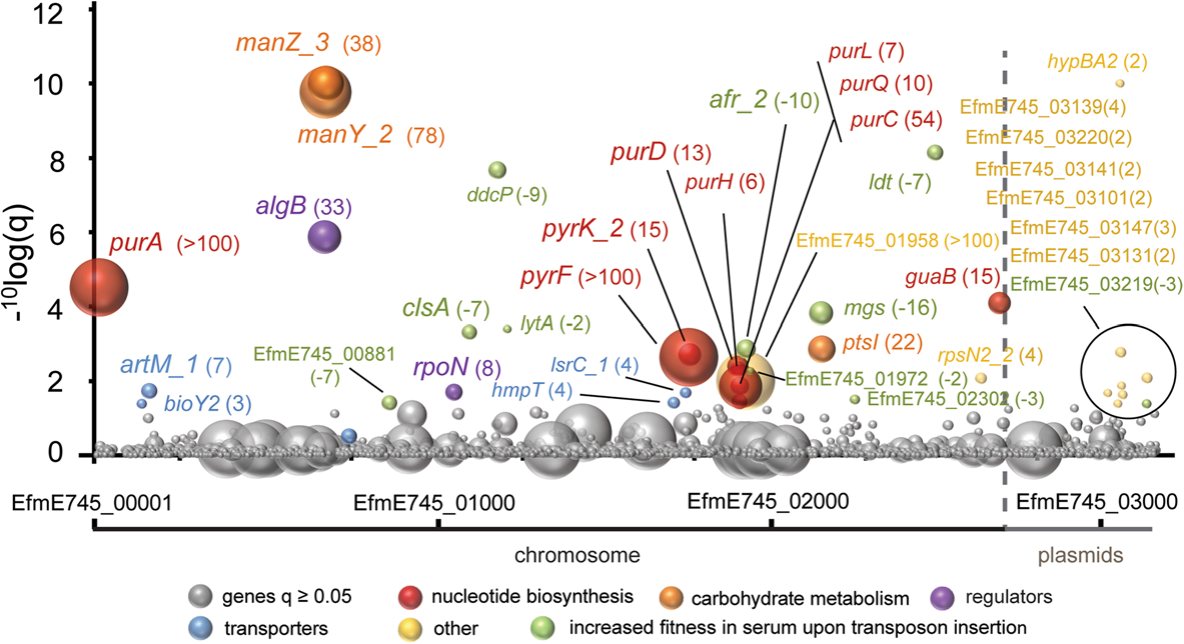
Tn-seq analysis to identify *E. faecium* genes required for growth in human serum. Bubbles represent genes, and bubble size corresponds to the fold-changes (for visual reasons, a 100-fold change in transposon mutant abundance is set as a maximum) derived from the read-count ratio of libraries grown in BHI to libraries grown in human serum. On the x-axis genes are shown in order of their genomic location and the chromosome and plasmids are indicated. The outcome of statistical analysis of the Tn-seq data is indicated on the y-axis. Genes with a significant change (q < 0.05) in fitness in serum versus BHI are grouped by function and are labelled with different colors, and the name or locus tag and the change in abundance between the control condition and growth in serum is indicated next to the bubbles in parentheses. Negative values indicate that mutants in these genes outgrow other mutants in serum, suggesting that these mutants, compared to the wild-type strain *E. faecium* E745, have a higher fitness in serum

We identified 37 genes that significantly contributed to growth of E745 in human serum (Fig. 3 and Additional file 8): twenty-nine genes were located on the chromosome and eight genes were present on plasmids (six genes on pE745–5, two genes on plasmid pE745–6). The relatively large number of genes identified indicates that growth of *E. faecium* in human serum is a multifactorial process. The genes that conferred the most pronounced effect on growth of *E. faecium* in serum included genes that are involved in carbohydrate uptake (*manZ_3, manY_2*, *ptsL*), a putative transcriptional regulator (*algB*) and genes involved in the biosynthesis of purine and pyrimidine nucleotides (*guaB*, *purA*, *pyrF*, *pyrK_2*, *purD*, *purH*, *purL*, *purQ*, *purC*) (Fig. 3). Notably, the *purD, purH,* and *purL* genes were found to exhibit higher expression upon growth in human serum in the RNA-seq analysis (Fig. 2). Nine genes were identified as negatively contributing to growth in serum, i.e. the transposon mutants in these genes were significantly enriched upon growth in serum. The effects of these mutations were relatively limited (Additional file 8), compared to the major effects observed in the transposon mutants discussed above, but it is notable that five (*clsA_1*, *ddcP, ldt*_*fm*_ *, mgs*, and *lytA_2*) of these genes have predicted roles in cell wall and cytoplasmic membrane biosynthesis.

**Fig. 4:**
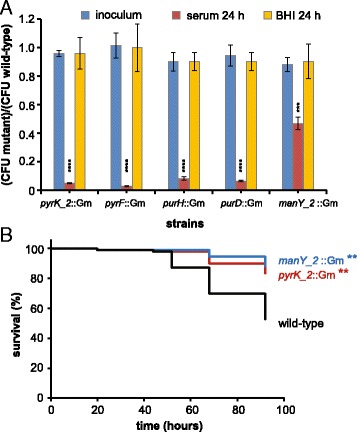
*E. faecium* transposon mutants with a growth defect in human serum and an attenuated phenotype in a zebrafish infection model. **a** Ratios of the viable counts of five mutants compared to wild-type *E. faecium* before (blue bars) and after 24 h of growth in human serum (red bars) or BHI (yellow bars). The viable counts of wild-type *E. faecium* E745 were (3.52 ± 0.07) × 10^5^/ml in the inocula, (2.92 ± 0.14) × 10^8^/ml after 24 h of growth in serum and (1.20 ± 0.20) × 10^9^/ml after 24 h of growth in BHI, respectively. Error bars represent the standard deviation of the mean of three independent experiments. Asterisks represent significant differences (***: *p* < 0.001, ****: *p* < 0.0001) as determined by a two-tailed Student’s *t*-test) between the mutant strains and wild-type. **b** Kaplan-Meier survival curves of zebrafish embryos upon infection with *E. faecium*. Infection was initiated by the injection of 1.2 × 10^4^ CFUs of the *manY_2*::Gm and *pyrK*::Gm transposon mutants and the wild-type *E. faecium* E745 into the circulation of zebrafish embryos 30 h post fertilisation*.* The experiment was performed three times and the mutants were significantly different (**: *p* < 0.01) from the wild-type in each experiment as determined by the Log-rank (Mantel-Cox) test with Bonferroni correction for multiple comparisons. This figure represents the combined results of the three replicates for *E. faecium* E745 (*n =* 93 zebrafish embryos), *manY_2*::Gm (*n =* 92) and *pyrK*::Gm (*n =* 90)

We developed a PCR-based method (Additional file 9) to selectively isolate five transposon mutants (in the purine metabolism genes *purD* and *purH*, the pyrimidine metabolism genes *pyrF* and *pyrK_2* and the phosphotransferase system (PTS) gene *manY_2* from the transposon library. Growth in rich medium of these transposon insertion mutants was equal to the parental strain. However, all mutants were significantly impaired in their growth in human serum (Fig. 4a), confirming the results of the Tn-seq experiments.

### *E. faecium pyrK_2* and *manY_2* contribute to intravenous infection of zebrafish

Finally, we investigated whether the transposon insertion mutants in the *manY_2* and *pyrK_2* genes were attenuated in vivo (Fig. 4b)*.* The mutants in these genes were selected because they represent the mutants in nucleotide and carbohydrate metabolism genes that were previously shown to contribute to the growth of *E. faecium* in human serum. As a model for intravenous infection, we used a recently described model in which *E. faecium* was injected into the circulation of zebrafish embryos to mimic systemic infections [37]. We showed that both the *manY_2* and the *pyrK_2* mutant were significantly less virulent than the parental strain. At 92 h post infection, survival of zebrafish embryos infected with the WT strain was 53%, as compared to 88% and 83% for zebrafish embryos that were infected with the transposon insertion mutants in *manY_2* and *pyrK_2*, respectively.

## Discussion

*E. faecium* can contaminate the skin and from there colonize indwelling devices such as intravenous catheters, or it can translocate from the gastrointestinal tract in immunosuppressed patients, leading to the development of bacteremia and endocarditis. *E. faecium* infections are often difficult to treat, due to the multi-drug resistant character of the strains causing nosocomial infections [3, 4]. However, the bloodstream poses challenges for the proliferation and survival of *E. faecium*, including a scarcity of nutrients.

In the present study, we sequenced the complete genome of a vancomycin-resistant *E. faecium* strain, and identified *E. faecium* genes that were essential for growth in human serum. A total of 37 genes, including genes with roles in carbohydrate uptake and nucleotide biosynthesis, were found to be required for fitness of *E. faecium* E745 in serum. Previously, fitness determinants for growth in human serum have been identified through large-scale screening of mutant libraries in both a Gram-negative (*Escherichia coli*) and a Gram-positive (*Streptococcus pyogenes*) pathogen [38, 39]. Notably, these studies have also identified the ability for de novo synthesis of purines and pyrimidines as a crucial factor for growth in serum. In addition, in diverse pathogenic bacteria (including *Burkholderia cepacia, Pasteurella multocida*, *Acinetobacter baumannii*, *Salmonella enterica* serovar Typhimurium, *Bacillus anthracis*, and *Streptococcus pneumoniae*), nucleotide biosynthesis contributes importantly to virulence [40–45]. The ability to synthesize nucleotides de novo thus appears to be an essential trait for the success of a pathogen that spreads through the bloodstream [38]. The data presented here indicate that de novo biosynthesis of nucleotides is also required for *E. faecium* growth in serum and virulence. The nucleotide biosynthesis pathway of *E. faecium* may be a promising target for the development of novel antimicrobials for the treatment of *E. faecium* bloodstream infections. Indeed, compounds that target guanine riboswitches, thereby inhibiting nucleotide biosynthesis, have already shown their efficacy in a *Staphylococcus aureus* infection model [46].

Three genes, *ptsL*, *manY_2* and *manZ_3*, encoding subunits of *E. faecium* PTSs were found to contribute to growth in serum in our Tn-seq experiments. The *ptsL* gene is predicted to encode an enzyme that confers a phosphate group from phosphoenolpyruvate to Enzyme I of PTS, while *manY_2* and *manZ_3* are predicted to form the IIA and IIBC components of a permease system that is homologous (64% and 69% amino acid identity, respectively) to the PtnAB PTS permease of *Lactococcus lactis* MG1363. PtnAB is one of the glucose uptake systems of *L. lactis* [47] and the *E. faecium* homolog may have a similar function, which could explain its essential role during growth in serum, as glucose is the only carbohydrate that occurs in the free state in appreciable amounts in serum [18].

It is notable that among the nine genes that exhibited increased fitness upon inactivation by transposon insertion, five genes are predicted to have a role in cell wall or cytoplasmic membrane biosynthesis. The protein encoded by *ddcP* was previously characterized as a low-molecular-weight penicillin-binding protein with D-alanyl-D-alanine carboxypeptidase activity [21], while *ldt*_*fm*_ acts as a peptidoglycan L,D transpeptidase [48]. The predicted α-monoglucosyldiacylglycerol synthase gene *mgs* is orthologous (73% amino acid identity) to *bgsB* in *E. faecalis*, which is required for the biosynthesis of membrane glycolipids [49]. The *clsA_1* gene is predicted to be responsible for the synthesis of cardiolipin (bisphosphatidylglycerol) and its inactivation may modulate the physical properties of the cytoplasmic membrane [50]. Finally, *lytA_2* is predicted to encode an autolysin, which may be involved in the turnover of peptidoglycan in the cell wall [51]. The transposon mutants in these genes were not further characterized in this study, but our findings suggest that non-essential pathways of cell wall or cytoplasmic membrane remodelling may confer subtle fitness defects to *E. faecium* when growing in a nutrient-poor environment, like serum.

Our RNA-seq-based transcriptional profiling of *E. faecium* E745 during mid-exponential growth in serum showed pervasive changes in gene expression compared to exponential growth in rich medium. The large number of differentially expressed genes may not all reflect the different growth conditions (serum and BHI) per se, but could also be influenced by the difference in growth rate during mid-exponential growth in serum and BHI (Additional file 2). The purine metabolism genes *purL*, *purH*, *purD,* which were found to be required for growth in serum in our Tn-seq experiments, were among those that were significantly upregulated during growth in serum compared to growth in rich medium. Notably, a single prophage was expressed at higher levels during growth in serum than in rich medium. The abundance of prophage elements in the genome of *E. faecium* has been noted before [4, 52]. Interestingly, in the related bacterium *Enterococcus faecalis* prophages encode platelet-binding proteins [53] and may have a role in intestinal colonization [54]. The contribution of *E. faecium* prophages to traits that are important for colonization and infection may provide important insights into the success of *E. faecium* as a nosocomial pathogen.

## Conclusions

Our data indicate that nucleotide biosynthesis and carbohydrate metabolism are critical metabolic pathways for the proliferation and survival of *E. faecium* in the bloodstream. The proteins encoded by the genes required for growth in human serum that were identified in this study, could serve as candidates for the development of novel anti-infectives for the treatment of bloodstream-infections by multi-drug resistant *E. faecium*.

## Methods

### Bacterial strains, plasmids, growth conditions, and oligonucleotides

The vancomycin-resistant *E. faecium* strain E745 was used throughout this study. This strain was isolated from a rectal swab of a hospitalized patient, during routine surveillance of a VRE outbreak in a Dutch hospital [27, 28]. Unless otherwise mentioned, *E. faecium* was grown in brain heart infusion broth (BHI; Oxoid) at 37 °C. The *E. coli* strains DH5*α* (Invitrogen) was grown in Luria-Bertani (LB) medium. When necessary, antibiotics were used at the following concentrations: chloramphenicol 4 μg ml^−1^ for *E. faecium* and 10 μg ml^−1^ for *E. coli*, and gentamicin 300 μg ml^−1^ for *E. faecium* and 25 μg ml^−1^ for *E. coli*. All antibiotics were obtained from Sigma-Aldrich. Growth was determined by measuring the optical density at 660 nm (OD_660_). The sequences of all oligonucleotides used in this study are listed in Additional file 10.

### Genome sequencing, assembly and bioinformatic analysis

*E. faecium* E745 was sequenced using a combination of Illumina HiSeq 100 bp paired-end sequencing, long-read sequencing using the Pacific Biosciences RS II SMRT technology and the MinION system with R7 flowcell chemistry (Oxford Nanopore Technologies). Corrected PacBio reads were assembled using the Celera assembler (version 8.1) [55] and assembled contigs were then corrected by aligning Illumina reads using BWA (version 0.7.9a), with default parameters for index creation and the BWA-MEM algorithm with the *-M* option for the alignment [56]. This approach resulted in 15 contigs, including one contig covering the entire 2.77 Mbp chromosome. After discarding contigs with low-coverage, the remaining contigs constituted 5 circular plasmid sequences and 5 non-overlapping contigs. These 5 contigs were aligned against the NCBI Genbank database and all were found to be part of the *E. faecium* plasmid pMG1 [57]. Based on this alignment the presumed order of contigs was determined and confirmed by gap-spanning PCRs and sequencing of the products. A single gap between two contigs, could not be closed by PCR. Thus, we assembled Illumina reads together with MinION 2D reads using the SPAdes assembler (version 3.0) [58], which produced a contig that closed the gap, resulting in a complete assembly of this plasmid. Sequence coverage of chromosomal and plasmid sequences was determined with SAMtools (version 0.1.18) using short read alignments to the assembly, which were generated using BWA (version 0.7.9a). SAMtools was also used to identify possible base-calling and assembly errors, by aligning short reads to assembled contigs. A base was corrected using the consensus of aligned reads [59]. The corrected sequences were annotated using Prokka (version 1.10) [60]. A maximum likelihood phylogenetic tree based on the core genome of *E. faecium* E745 and an additional 72 *E. faecium* strains representing the global diversity of the species [4], was generated using ParSNP [61] with settings -c (forcing inclusion of all genome sequences) and -x (enabling recombination detection and filtering). The resulting phylogenetic tree was visualized using MEGA 7.0.26 [62]. Antibiotic resistance genes in the assembled genome sequence of *E. faecium* E745 were identified using ResFinder [63]. The annotated genome of *E. faecium* E745 is available from NCBI Genbank database under accession numbers CP014529 – CP014535.

### RNA-seq

Approximately 3 × 10^7^ cfu of *E. faecium* E745 were inoculated into 14 ml of BHI broth and heat-inactivated serum, and grown at 37 °C until exponential phase. Cultures were centrifuged at room temperature (15 s; 21.380 *g*), and pellets were flash frozen in liquid N_2_ prior to RNA extraction, which was performed as described previously [21]. The ScriptSeq Complete Kit (Bacteria) (Epicentre Biotechnologies, WI) was used for rRNA removal and strand-specific library construction. Briefly, rRNA was removed from 2.5 μg of total RNA. To generate strand specific RNA-seq data, approximately 100 ng of rRNA-depleted RNA was fragmented and reverse transcribed using random primers containing a 5′ tagging sequence, followed by 3′ end tagging with a terminal-tagging oligo to yield di-tagged, single-stranded cDNA. Following magnetic-bead based purification, the di-tagged cDNA was amplified by PCR (15 cycles) using ScriptSeq Index PCR Primers (Epicentre Biotechnologies, WI). Amplified RNA-seq libraries were purified using AMPure XP System (Beckman Coulter) and sequenced by a 100 bp paired end reads sequencing run using the Illumina HiSeq 2500 platform (University of Edinburgh, United Kingdom). Data analysis was performed using Rockhopper [64] using the default settings for strand specific analysis.

### Validation of RNA-seq results by quantitative real-time RT-PCR (qRT-PCR).

Total RNA isolated as described previously was used to confirm the transcriptome analysis by qRT-PCR. cDNA was synthesized as described above and qRT-PCR on these cDNAs was performed using the Maxima SYBR Green/ROX qPCR Master Mix (Thermo Scientific, Breda, The Netherlands) and a StepOnePlus instrument (Life Technologies). The expression of *tufA* was used as a housekeeping control. Ct values were calculated using the StepOne analysis software v2.2. Transcript levels, relative to *tufA,* of the assayed genes were calculated using REST 2009 V2.0.13 (Qiagen, Venlo, the Netherlands). This experiment was performed with three biological replicates.

### Generation of *mariner* transposon mutant library in *E. faecium*

To create a transposon mutant library in *E. faecium* E745 suitable for Tn-seq, the *mariner* transposon cassette (carrying a gentamicin resistance gene) in the transposon delivery plasmid pZXL5 [21] was adapted as follows. The transposon from pZXL5 was amplified by PCR using the set of primers: pZXL5_MmeI_SacII_Fw and pZXL5_MmeI_SacII_Rv. These primers introduced MmeI restriction sites in the inverted repeats on both sides of the transposon. The modified transposon delivery vector, termed pGPA1, was generated by the digestion of pZXL5 with SacII, followed by the insertion of the SacII-digested *mariner* transposon that contained MmeI restriction sites at its extreme ends. pGPA1 was electroporated into *E. faecium* E745 and the transposon mutant library was generated by selecting for gentamicin-resistant transposon mutants as described previously [21].

### Tn-seq analysis of conditionally essential genes in *E. faecium* E745

The transposon mutant library created in E745 was prepared for Tn-seq analysis, similar to previously described procedures [65]. To identify genes that are essential for the viability of *E. faecium* in BHI*,* we used ten experimental replicates of the mutant library. Aliquots (20 μl) of the transposon mutant library, containing approximately 10^7^ cfu, were used to inoculate 20 ml BHI broth and grown overnight at 37 °C. Subsequently, 1 ml aliquots of the cultures were spun down (15 s, 21.380 *g*) and used for the extraction of genomic DNA (Wizard genomic DNA purification kit, Promega Benelux). 2 μg of the extracted DNA was digested for 4 h at 37 °C using 10 U MmeI (New England Biolabs) and immediately dephosphorylated with 1 U of calf intestine alkaline phosphatase (Invitrogen) during 30 min at 50 °C. DNA was isolated using phenol-chloroform extraction and subsequently precipitated using ethanol. The DNA pellets were then dissolved in 20 μl water. The samples were barcoded and prepared for Tn-seq sequencing as described previously [65]. The sequence reads from all ten experimental replicates were mapped to the genome, and the mapped read-counts were then tallied for the analysis of the essentiality of the genes in the *E. faecium* E745 genome (further described below).

To identify genes that are required for growth in human serum, 20 μl aliquots of the frozen mutant library in E745 were inoculated in BHI broth and grown overnight as described above. Subsequently, bacterial cells were washed with physiological saline solution. Approximately 3 × 10^7^ cfu were inoculated into 14 ml BHI broth, and approximately 3 × 10^6^ cfu were inoculated into 14 ml human serum obtained from Sigma (Cat. No. H4522; Sterile filtered type-AB human serum) or heat-inactivated human serum (the same, after incubation for 30 min at 56 °C). The different inoculum-sized were used in order for a similar number of divisions to occur during the experiment. Cells were incubated at 37 °C for 24 h without shaking and then further processed for Tn-seq [65]. This experiment was performed in triplicate.

Tn-seq samples were sequenced (50 nt, single-end) on one lane of a Illumina Hiseq 2500 (Baseclear, Leiden, the Netherlands and Sequencing facility University Medical Center, Utrecht, The Netherlands), generating an average of 15 million high quality reads per sample.

### Tn-seq data analysis

Raw Illumina sequence reads from Illumina sequencing were split, based on their barcode, using the Galaxy platform [66], and 16-nucleotide fragments of each read that corresponded to E745 sequences, were mapped to the E745 genome using Bowtie 2 [67]. The results of the alignment were sorted and counted by IGV [68] using a 25-nucleotide window size and then summed over the gene. Read mapping to the final 10% of a gene were discarded as these insertions may not inactivate gene function. Read counts per gene were then normalized to the total number of reads that mapped to the genome in each replicate, by calculating the normalized read-count RPKM (Reads Per Kilobase per Million input reads) via the following formula: RPKM = (number of reads mapped to a gene × 10^6^) / (total mapped input reads in the sample x gene length in kbp). Statistical analysis of the RPKM-values between the experimental conditions was performed using Cyber-T [69]. Genes were determined to be significantly contributing to growth in human serum when the Benjamini-Hochberg corrected *P-*value was <0.05 and the difference in abundance of the transposon mutant during growth in BHI and serum was >2.

### Isolation of mutants from the transposon mutant library pool

To recover a targeted transposon mutant from the complete mutant pool, a PCR-based screening strategy was developed (Additional data file 9). 40 μl of the transposon mutant library was inoculated into 40 ml of BHI broth with gentamicin and grown overnight at 37 °C with shaking (200 rpm). The overnight culture, containing approximately 10^9^ cfu/ml, was then diluted to approximately 20 cfu/ml in 500 ml of BHI with gentamicin and kept on ice. Subsequently, 200 μl aliquots were transferred to wells of sterile 96 wells plates (*n* = 12, Corning Inc.). After overnight incubation at 37 °C without shaking, aliquots (15 μl) of each one of the 96 wells, were further pooled into a single new 96 well plate, as described in Additional file 9.

PCRs were performed on the final plate in which the transposon mutants were pooled, to check for the presence of the Tn-mutants of interest, using the primer ftp_tn_both_ends_MmeI, which is complementary to the repeats flanking the transposon sequence, in combination with a gene-specific primer. When a PCR was found to be positive in one of the wells of this plate, the location of the Tn-mutant was tracked backwards to the wells containing approximately 4 independent transposon mutants, by performing PCRs mapping the presence of the transposon mutant in each step. Cells from the final positive well were plated onto BHI with gentamicin and colony PCR was performed to identify the desired transposon mutant.

### Growth of *E. faecium* E745 and individual mutants in human serum

Wild-type E745 and the mutant strains were grown overnight at 37 °C in BHI broth. Subsequently, bacterial cells were washed with physiological saline and approximately 3 × 10^5^ cfu were inoculated into 1.4 ml BHI broth or heat-inactivated serum. Cells were grown in 1.5 ml tubes (Eppendorf) in triplicate for each condition and incubated at 37 °C for 24 h without shaking. Bacterial growth was determined by assessing viable counts, for which the cultures were serially diluted using physiological saline solution and plated onto BHI agar followed by overnight incubation at 37 °C.

### Intravenous infection of zebrafish embryos

London wild-type (LWT) inbred zebrafish embryos, provided by the aquarium staff of The Bateson Center (University of Sheffield), were used for infection experiments. The parental E745 strain and its *pyrK_2* and *manY_2* transposon mutants were grown in BHI broth until they reached an optical density at 600 nm of approximately 0.5 and were then harvested by centrifugation (5500 *g*, 10 min). Bacteria were microinjected into the circulation of dechorionated zebrafish embryos at 30 h post fertilization, as previously described [70]. Briefly, anesthetized embryos were embedded in 3% (*w*/*v*) methylcellulose and injected individually with approximately 1.2 × 10^4^ cfu using microcapillary pipettes. For each strain, 29 to 32 infected embryos were observed for survival up to 90 h post infection (hpi). This experiment was performed in triplicate.

## Additional files

Additional file 1: Genome sequence information for *E. faecium* E745. (XLSX 10 kb)

Additional file 2: Growth of *E. faecium* E745 in BHI and serum. (PDF 962 kb)

Additional file 3: Operons identified by RNA-seq in *E. faecium* E745. (XLSX 40 kb)

Additional file 4: *E. faecium* E745 genes that exhibited significant (q < 0.001 and fold-change > 2) differential expression in human serum, as determined by RNA-seq. (XLSX 70 kb)

Additional file 5: qRT-PCR validation of RNA-seq experiments. Correlation of RNA-seq and qRT-PCR expression ratios for the seven genes with various expression levels and genomic locations. The gene expression ratios obtained from both qRT-PCR and RNA-seq were normalized by a housekeeping control gene Efm745_00056 (*tufA*). The experiment was performed with three biological replicates. (PDF 121 kb)

Additional file 6: Characterization of the *E. faecium* E745 transposon mutant library, showing the number of reads that were mapped to the *E. faecium* E745 chromosome and plasmids. The height of each peak represents the read abundance at a specific insertion site. On the y-axis, the number of mapped reads is shown on a log scale. (PDF 1427 kb)

Additional file 7: Tn-seq data: comparison of heat-inactivated and native serum (XLSX 139 kb)

Additional file 8: *E. faecium* E745 genes that significantly (q < 0.05 and fold-change <−2 or >2) contribute to growth in human serum, as identified by Tn-seq. (XLSX 13 kb)

Additional file 9: Isolation of mutants from the transposon mutant library pool. (A) Schematic representation of the PCR reaction designed to find a particular Tn-mutant within the transposon mutant library. This PCR uses a combination of a gene-specific primer (blue arrow) and a transposon specific primer primer (yellow arrow). Positive PCR products, indicated by the green check marks, should occur when the transposon (depicted as a yellow triangle) is inserted in the gene of interest (depicted in blue). If the transposons inserted in adjacent genes or intergenic regions, no PCR product can be amplified (red crosses). (B) Schematic workflow to isolate Tn-mutants from the mutant library. The transposon mutant library is split into 12 plates (A1 - A12) of 96 wells, with each 200 μl well containing an average of 4 mutants. Plates were incubated overnight (Step 2). Plate A1 was then pooled into the first column of a new 96 well plate, denominated plate B1 (Step 3) and the same was done for plates A2 to A12. Subsequently, plate B1 was pooled again into the first column of a third plate, denominated C1 (Step 4). PCR using the gene-specific primer and the transposon specific primer was performed on the 8 wells of plate C1 (Step 5). A positive PCR was suggestive of the presence of a particular transposon-mutant (depicted as a red dot). The presence of the transposon mutant was then confirmed by PCR in plate B1 (step 6) and the corresponding plate A (step 7). Once a transposon-mutant was located to a particular well in plate A, the well was plated on BHI plates containing gentamicin, and the colonies were screened for the presence of the transposon mutant by PCR (step 8). (PDF 277 kb)

Additional file 10: Oligonucleotides used in this study. (XLSX 10 kb)

## Abbreviations

BHI: Brain Heart Infusion broth
cDNA: complementary DNA
cfu: colony forming units
hpi: hours post-infection
kbp: kilo-base pair
LB: Luria-Bertani
LWT: London wild-type
NCBI: National Center for Biotechnology Information
nt: nucleotides
PTS: phosphotransferase system
qPCR: quantitative polymerase chain reaction
RNA-seq: RNA sequencing
RPKM: reads per kilobase per million input reads
rRNA: ribosomal RNA
Tn-seq: transposon sequencing
TraDIS: transposon directed insertion sequencing
U: unit(s)
