# Supplementary material for: RNA-seq and Tn-seq reveal fitness determinants of vancomycin-resistant Enterococcus faecium during growth in human serum
Source: BMC Genomics. 2017 Nov 21;18:893. doi: 10.1186/s12864-017-4299-9 (PMC5699109; doi:10.1186/s12864-017-4299-9)
Supplement: Supplementary file 2 — Growth of E. faecium E745 in BHI and serum. (PDF 962 kb) [file 12864_2017_4299_MOESM2_ESM.pdf]

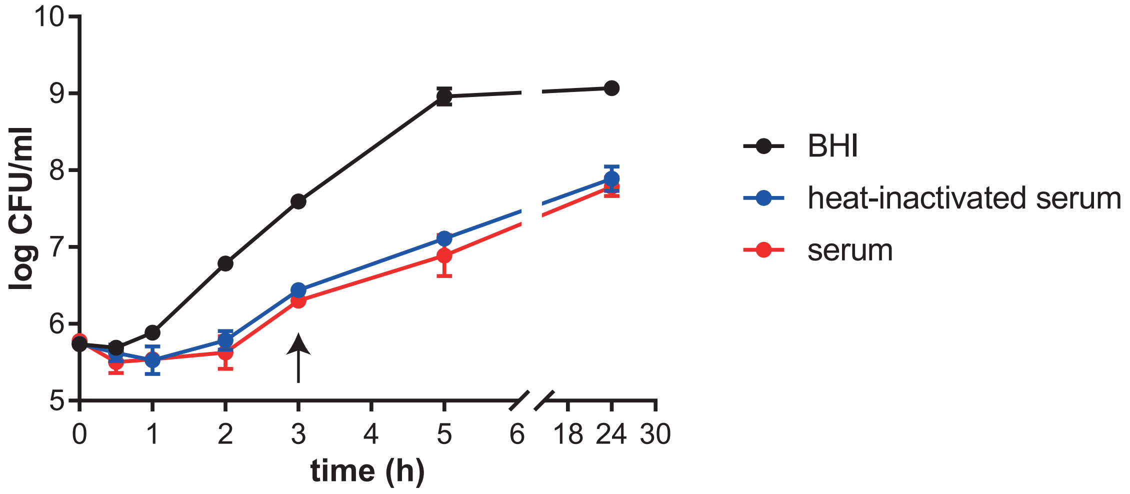

Additional file 2. Growth curves of E. faecium E745 in Brain Heart Infusion (BHI) broth, heat-inactivated and native serum at 37°C. The results are the average of three independent experiments for BHI, and two independent experiments for the sera. Error bars indicate standard deviations. The arrow corresponds to the time of sampling for the RNA-seq experiments.
