# Supplementary material for: RNA-seq and Tn-seq reveal fitness determinants of vancomycin-resistant Enterococcus faecium during growth in human serum
Source: BMC Genomics. 2017 Nov 21;18:893. doi: 10.1186/s12864-017-4299-9 (PMC5699109; doi:10.1186/s12864-017-4299-9)
Supplement: Supplementary file 5 — qRT-PCR validation of RNA-seq experiments. Correlation of RNA-seq and qRT-PCR expression ratios for the seven genes with various expression levels and genomic locations. The gene expression ratios obtained from both qRT-PCR and RNA-seq were normalized by a housekeeping control gene Efm745_00056 (tufA). The experiment was performed with three biological replicates. (PDF 121 kb) [file 12864_2017_4299_MOESM5_ESM.pdf]

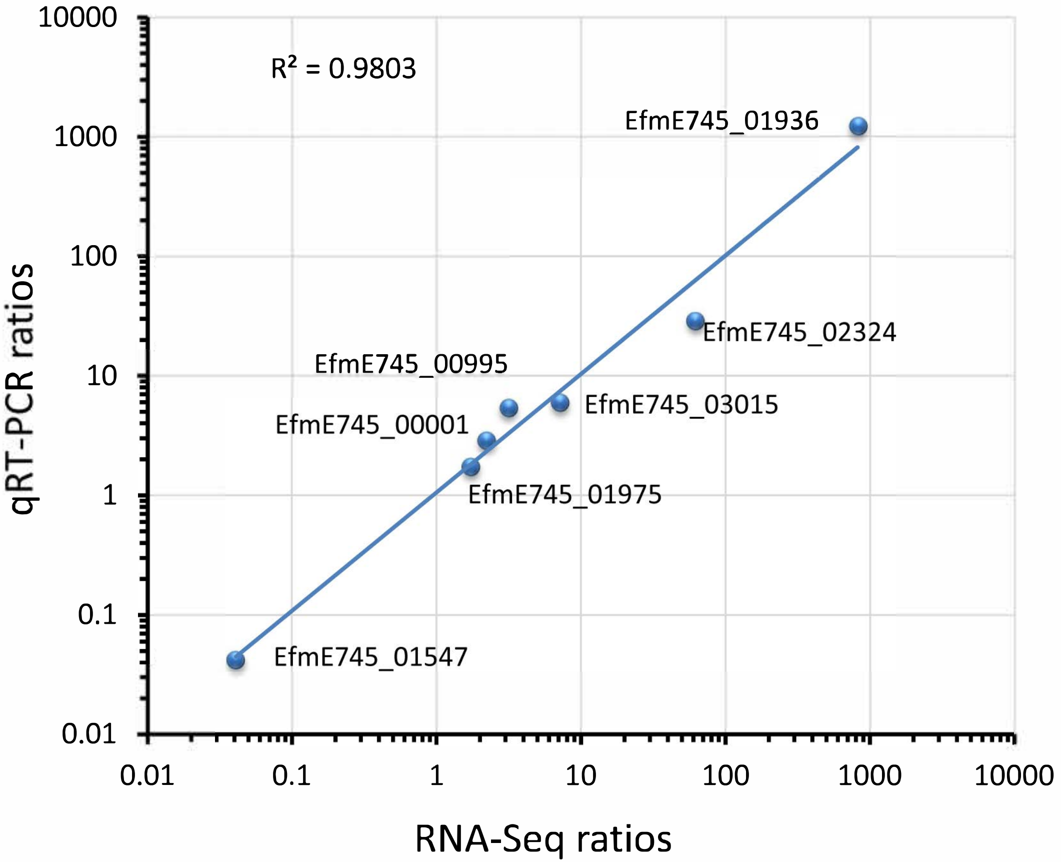


**Additional file 5. qRT-PCR validation of RNA-Seq experiments.** Correlation of RNA-Seq and qRT-PCR expression ratios for the seven genes with various expression levels and genomic locations. The gene expression ratios obtained from both qRT-PCR and RNA-Seq were normalized by a housekeeping control gene Efm745_00056 (*tufA*). The experiments was performed with three biological replicates.
