# Supplementary material for: RNA-seq and Tn-seq reveal fitness determinants of vancomycin-resistant Enterococcus faecium during growth in human serum
Source: BMC Genomics. 2017 Nov 21;18:893. doi: 10.1186/s12864-017-4299-9 (PMC5699109; doi:10.1186/s12864-017-4299-9)
Supplement: Supplementary file 6 — Characterization of the E. faecium E745 transposon mutant library, showing the number of reads that were mapped to the E. faecium E745 chromosome and plasmids. The height of each peak represents the read abundance at a specific insertion site. On the y-axis, the number of mapped reads is shown on a log scale. (PDF 1427 kb) [file 12864_2017_4299_MOESM6_ESM.pdf]

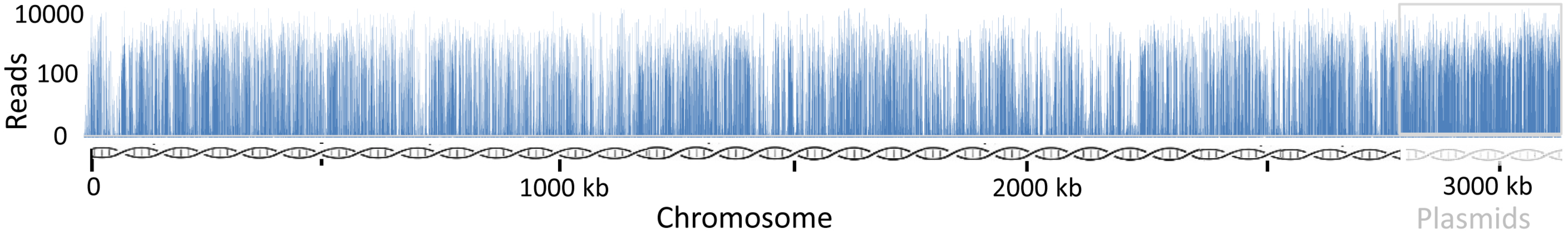

Additional file 6. Characterization of the E. faecium E745 transposon mutant library, showing the number of reads that were mapped to the E. faecium E745 chromosome and plasmids. The height of each peak represents the read abundance at a specific insertion site. On the y-axis, the number of mapped reads are shown in log scale.
