# Supplementary material for: RNA-seq and Tn-seq reveal fitness determinants of vancomycin-resistant Enterococcus faecium during growth in human serum
Source: BMC Genomics. 2017 Nov 21;18:893. doi: 10.1186/s12864-017-4299-9 (PMC5699109; doi:10.1186/s12864-017-4299-9)
Supplement: Supplementary file 9 — Isolation of mutants from the transposon mutant library pool. (A) Schematic representation of the PCR reaction designed to find a particular Tn-mutant within the transposon mutant library. This PCR uses a combination of a gene-specific primer (blue arrow) and a transposon specific primer primer (yellow arrow). Positive PCR products, indicated by the green check marks, should occur when the transposon (depicted as a yellow triangle) is inserted in the gene of interest (depicted in blue). If the transposons inserted in adjacent genes or intergenic regions, no PCR product can be amplified (red crosses). (B) Schematic workflow to isolate Tn-mutants from the mutant library. The transposon mutant library is split into 12 plates (A1 - A12) of 96 wells, with each 200 μl well containing an average of 4 mutants. Plates were incubated overnight (Step 2). Plate A1 was then pooled into the first column of a new 96 well plate, denominated plate B1 (Step 3) and the same was done for plates A2 to A12. Subsequently, plate B1 was pooled again into the first column of a third plate, denominated C1 (Step 4). PCR using the gene-specific primer and the transposon specific primer was performed on the 8 wells of plate C1 (Step 5). A positive PCR was suggestive of the presence of a particular transposon-mutant (depicted as a red dot). The presence of the transposon mutant was then confirmed by PCR in plate B1 (step 6) and the corresponding plate A (step 7). Once a transposon-mutant was located to a particular well in plate A, the well was plated on BHI plates containing gentamicin, and the colonies were screened for the presence of the transposon mutant by PCR (step 8). (PDF 277 kb) [file 12864_2017_4299_MOESM9_ESM.pdf]

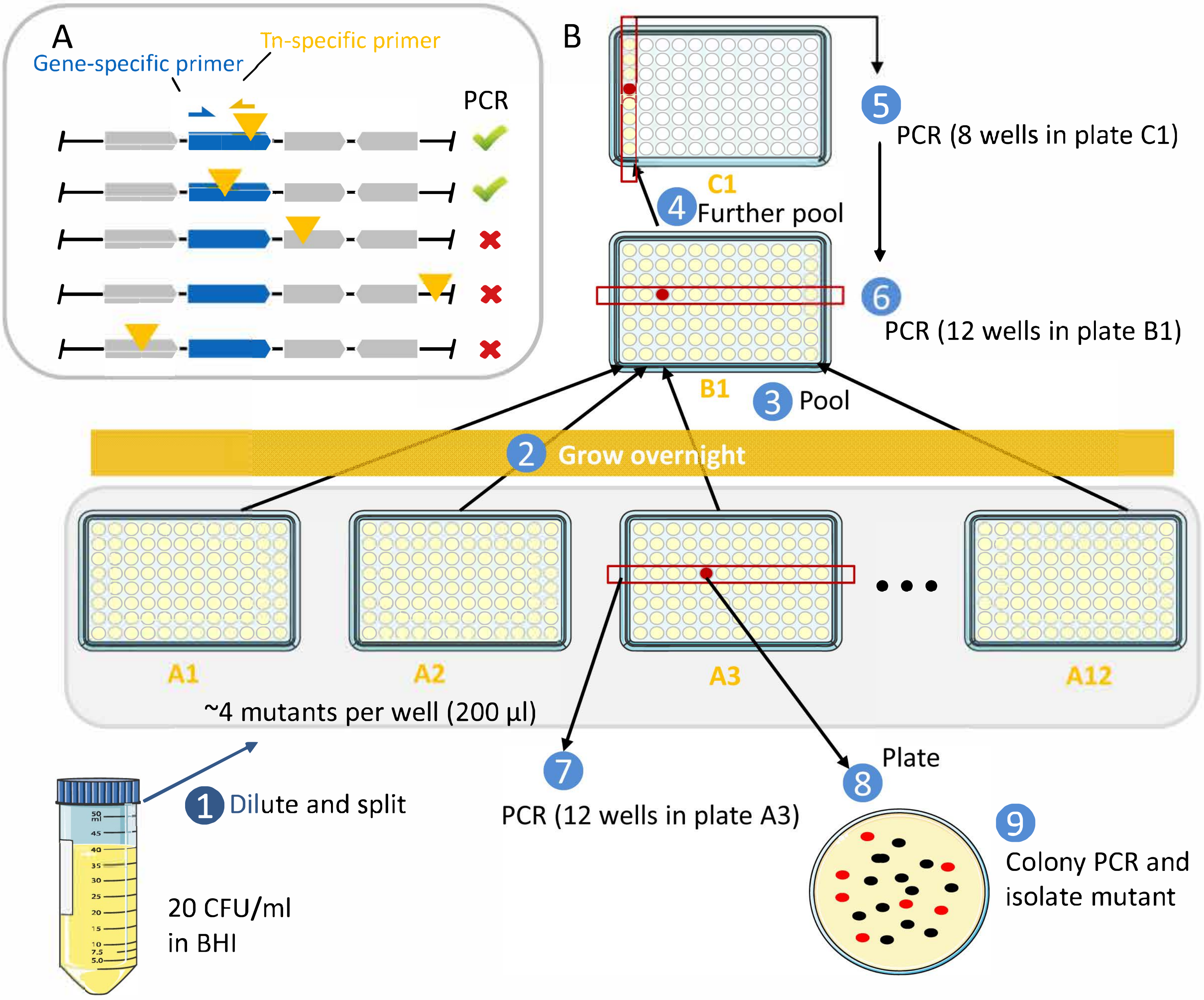

Additional file 9. Isolation of mutants from the transposon mutant library pool. (A) Schematic representation of the PCR reaction designed to find a particular Tn-mutant within the transposon mutant library. This PCR uses a combination of a gene-specific primer (blue arrow) and a transposon specific primer primer (yellow arrow). Positive PCR products, indicated by the green check marks, should occur when the transposon (depicted as a yellow triangle) is inserted in the gene of interest (depicted in blue). If the transposons inserted in adjacent genes or intergenic regions, no PCR product can be amplified (red crosses). (B) Schematic workflow to isolate Tn-mutants from the mutant library. The transposon mutant library is split into 12 plates (A1 - A12) of 96 wells, with each 200 µl well containing an average of 4 mutants. Plates were incubated overnight (Step 2). Plate A1 was then pooled into the first column of a new 96 well plate, denominated plate B1 (Step 3) and the same was done for plates A2 to A12. Subsequently, plate B1 was pooled again into the first column of a third plate, denominated C1 (Step 4). PCR using the gene-specific primer and the transposon specific primer was performed on the 8 wells of plate C1 (Step 5). A positive PCR was suggestive of the presence of a particular transposon-mutant (depicted as a red dot).The presence of the transposon mutant was then confirmed by PCR in plate B1 (step 6) and the corresponding plate A (step 7). Once a transposon-mutant was located to a particular well in plate A, the well was plated on BHI plates containing gentamicin, and the colonies were screened for the presence of the transposon mutant by PCR (step 8).
